# Subgroups of Children with Autism Spectrum Disorder without Intellectual Disability: A Longitudinal Examination of Executive and Socio-Adaptive Behaviors in Adolescence

**DOI:** 10.3390/jcm10102220

**Published:** 2021-05-20

**Authors:** Rocio Rosello, Carmen Berenguer, Jose Martinez-Raga, Ana Miranda, Samuele Cortese

**Affiliations:** 1 Division of Psychiatry, Imperial College London, London W12 0NN, UK; 2 Division of Psychiatry, University Hospital Doctor Peset of Valencia, 46017 Valencia, Spain; martinez_josrag@gva.es; 3 Division of Developmental and Educational Psychology, University of Valencia, 46010 Valencia, Spain; carmen.berenguer@uv.es (C.B.); ana.miranda@uv.es (A.M.); 4 Division of Psychiatry, University of Valencia, 46010 Valencia, Spain; 5 Hassenfeld Children’s Hospital at NYU Langone, New York University Child Study Center, New York, NY 10016, USA; samuele.cortese@gmail.com; 6 Child and Adolescent Mental Health Service, Solent NHS Trust, Southampton SO19 6DR, UK; 7 Centre for Innovation in Mental Health (CIMH), School of Psychology, Faculty of Environmental and Life Sciences, University of Southampton, Southampton SO17 1BJ, UK; 8 Division of Psychiatry and Applied Psychology, School of Medicine, University of Nottingham, Nottingham NG7 2RD, UK

**Keywords:** autism subgroups, adolescents, executive functioning, social skills, adaptive behavior

## Abstract

Within the autistic spectrum, there is remarkable variability in the etiology, presentation, and treatment response. This prospective study was designed to identify, through cluster analysis, subgroups of individuals with ASD without intellectual disability (ID) based on the severity of the core symptoms in childhood. The secondary aim was to explore whether these subgroups and a group with typical development (TD) differ in cognitive, adaptive, and social aspects measured in adolescence. The sample at baseline was comprised of 52 children with ASD without ID and 37 children with TD, aged 7–11. Among the ASD group, three clusters were identified. Cluster 1 (40%), ‘high severity’, presented high symptom severity on the DSM-5 criteria and the Social Communication Questionnaire. Cluster 2 (34%) showed ‘moderate severity’ on most of the scores. Cluster 3 (25%) corresponded to ‘low severity’, showing moderate social impairment and low restrictive, repetitive patterns of behavior, interests and activities. At 5-year follow-up, 45 adolescents with ASD without ID and 27 adolescents with TD were assessed. All clusters had significantly more difficulties in EF, ToM, socialization and adaptive behavior compared to TD. Social and adaptive trajectories between the ASD subgroups were relatively different; Cluster 3 showed poorer socialization and daily living skills than the other two subgroups. These findings highlight the importance of fully assessing social, cognitive, and adaptive profiles to develop care plans tailored to specific needs.

## 1. Introduction

Autism spectrum disorder (ASD) is conceptualized in the latest edition of the Diagnostic and Statistical Manual of Mental Disorders (DSM-5; American Psychiatric Association, 2013) [1] as a single diagnostic category with impairments in two dimensions, social communication and restricted, repetitive patterns of behavior, interests, and activities (RRBI). However, within the autistic spectrum, there is remarkable variability in the etiology, presentation, and treatment response. In order to enhance phenotypic homogeneity, previous research has used cluster analysis to develop meaningful ASD subgroups based on empirical characteristics that may reflect clinical phenotypes. 

### 1.1. Cluster Studies in Children with ASD, with and without Intellectual Disability

The identification of clusters in groups of children with ASD has mainly been based on the severity of the core symptoms, assessed with the Autism Diagnostic Observation Schedule (ADOS) [2] and the Autism Diagnostic Interview—Revised (ADI-R) [3]. The majority of the subtyping studies have included children with ASD with a broad Intellectual Quotient (IQ) range, DSM-IV [4], or DSM-5 [1], identifying cluster solutions consisting of three subgroups: one with all the autism symptoms severely affected; another presenting moderate socio-communicative impairment (SCI), but less RRBI; and a third subgroup characterized by lower SCI and relatively more RRBI [5,6]. Along similar lines, Zheng et al. [7] highlighted three clusters: a subgroup of children with relatively high language, cognitive, and adaptive abilities, along with relatively low RRB and social difficulties; another cluster characterized by similar language, cognitive, and adaptive abilities, but more RRB and social symptoms; and a third cluster with the highest levels of impairment in the developmental areas explored. 

Intellectual functioning may be crucial in exploring the variability in the behavioral expression of ASD phenotypes. Surprisingly, few investigations focusing on phenotypic characteristics have identified potential subgroups of children with ASD without intellectual disability (ID), even though they represent approximately two-thirds of the ASD population [8]. Importantly, it is unknown to what extent the cognitive and social deficits seen in ASD are expressed as a function of the severity of the symptomatology of the disorder. The study by Bitsika et al. [9] provided two clusters, ‘high severity’ and ‘low severity’, each involving different levels of social-communicative and restricted-repetitive behavioral deficits. More clearly, the two clusters obtained by Klopper et al. [10] were separated according to the two blocks of ASD symptoms. The first profile had more social and communicative problems and few repetitive behaviors, whereas the second profile showed better social and communicative skills, but numerous repetitive behaviors. Several studies through cluster analysis using language measures have also found two subgroups. Harper-Hill et al. [11], with scores of spoken nonword repetition and sentence repetition, identified two clusters within a group of ASD and TD children (mean age 11.4, IQ: 100). Cluster 1 presented poorer linguistic performance and a tendency towards higher severity of autistic symptoms than Cluster 2. Tanaka et al. [12] obtained similar results from a cluster analysis using the characteristics of communication of children (mean IQ: 105). One of the two subtypes was associated with low language competence and strong characteristics of autism, while the other was associated with relatively high language competence and milder characteristics of autism.

Zheng et al. [13], using only the scores on RRBI, distinguished three subgroups of children with ASD, labeled as high, medium, and low severity. These subgroups also differed on the level of their difficulties in adaptive behaviors, autistic traits, and problematic behaviors. Mira et al. [14] obtained three clusters taking into account children’s adaptive, behavioral, and pragmatic behaviors in a sample of children aged 7–11 (mean IQ: 101). Cluster 1 was characterized by ‘serious difficulties’, Cluster 3 had ‘slight difficulties’ and Cluster 2 showed ‘moderate difficulties’ in all the domains evaluated. The cluster with ‘slight difficulties’ was only significantly differentiated from the group with TD on symptoms of ASD and attention deficit hyperactivity disorder (ADHD), pragmatic competence, and applied theory of mind. In addition, mothers of children in the ‘serious difficulties’ cluster showed higher stress levels and less use of support and acceptance strategies, as well as more mental health problems, than mothers of children in the ‘slight difficulties’ cluster. Finally, a recent study by Brigido et al. [15] analyzed different profiles of ASD without ID in SCI and RRBI, considering both behavior type and frequency. The ASD typical behaviors were analyzed based on DSM-5 criteria and the ASD Typical Behaviors Questionnaire (ASD-TBQ) in a sample of 75 children aged between 8 and 12 years old. Two groups were identified which differed in ASD diagnostic domains and subdomains as well as most of the behaviors: Group 1 presented a lower frequency of behaviors, being higher in SCI, while Group 2 presented a similar frequency in both domains evaluated (SCI and RRBI).

### 1.2. Studies of Developmental Outcomes for Clusters of Children with ASD

Prospective research exploring the developmental trajectory of different clinical clusters of children with ASD is limited. Stevens et al. [16] identified two ASD subgroups—high functioning and low functioning—in preschoolers, based on expressive and receptive language abilities, non-verbal intelligence, and social behavior. Over time, at 7–9 years of age, the two clusters showed significant differences in terms of social impairment, language skills, and cognitive profiles, with a significant number of children in the high functioning subgroup achieving neurotypical scores in behavioral and cognitive domains. In contrast, abnormalities in social behavior, communication, and RRBI continued to be pronounced at follow-up in the group labeled as low functioning.

Brennan et al. [17], in another hierarchical classification of two-year-old children with ASD, detected three clusters: Cluster 1 had mild autism symptoms and high cognitive ability; Cluster 2 exhibited relatively more restricted and repetitive behaviors, social and communicative impairments, and low cognitive abilities; and Cluster 3 was characterized by severe social, communicative, and cognitive deficits, but with no repetitive behaviors. Follow-up of diagnostic stability at age four showed that 60% of the participants in Cluster 1 no longer met the criteria for the ASD diagnosis, whereas 89.5% of the children in Cluster 2 fulfilled the PDD criteria. Wiggins et al. [18] established three clinical clusters of ASD participants at age two, based on social, communicative, and cognitive abilities, along with repetitive behaviors and sensory features. Subgroup 1, ‘mild impairment’, had above-sample-average scores in all domains; the second subgroup, ‘moderate impairment’, showed high levels of social and communicative deficits, few RRBI, and mild intellectual disability. The third subgroup, ‘severe impairment’, had mild–moderate intellectual deficits and salient impairments across domains. At the two-year follow-up, no significant changes were observed in the characteristics of the clinical clusters; nevertheless, 19.17% of the children with a diagnosis of ASD at baseline no longer met the diagnostic criteria. Ausderau et al. [19] identified in children with ASD (ages 2–12 years) four differential sensory subtypes: mild, extreme-mixed, sensitive-distressed and attenuated-distressed. The subtypes had distinctive child (gender, developmental stage, chronological age, autism severity) and family (income, level of mother’s education) characteristics. Ninety-one percent of participants remained unchanged in their subtypes over 1 year.

Even though transitioning to adolescence is crucial as it can be particularly challenging for children with autism, no previous study has evaluated the cognitive and social behavior in adolescence of subgroups identified in childhood based on the severity of their symptoms. According to the two-hit model, the atypical neural system of individuals with ASD is less likely to adequately respond to an increase in social demands and acquire new prosocial behaviors [20]. Previous research supports the notion that, across many cognitive and social domains, developmental outcomes of children with ASD remain unchanged or decline during adolescence [21]. For many adolescents, building and maintaining age-appropriate peer relationships allows them to share experiences and feelings and learn how to solve conflicts, whereas social isolation reduces opportunities to develop social skills. Adolescents with ASD with and without ID have fewer real friends and meet their friends less often outside school compared to their peers [22]. Furthermore, the association between ASD symptoms and poor prosocial behavior in adolescence is highly stable [23]. Several factors could be involved. On the one hand, Theory of Mind (ToM)—the ability to share feelings, exchange ideas, and anticipate others’ behavior—is one of the factors that could explain the socialization development of children with ASD without ID [24]. Likewise, in adolescence, poor ToM skills are associated with more severe SCI and RRBI [25], and they help to discriminate among severity levels, that is, severe, moderate, and mild [26]. On the other hand, deficits in social communication skills, such as failing to engage in conversations and read non-verbal cues, as well as RRBI and limited flexibility, may lead to exclusion from peer groups. Moreover, deficits in executive functioning (EF) are exhibited by individuals with ASD, regardless of their age and level of intelligence, with flexibility, inhibition of an inappropriate response, and planning being the most commonly impaired [27,28,29]. In fact, there is an association between informants’ rating of EF impairments in behavioral regulation [30] and metacognition [31] with poor real-world social behaviors in individuals with ASD. Metacognitive skills and behavioral regulation components differentially impact social functioning in children with ASD and in children with TD. In particular, behavioral regulation processes such as inhibition, emotional control and shifting have predictive power on social functioning in all children, while metacognitive processes predict social functions only in children with ASD [30]. More specifically, monitoring skills predict social awareness, shifting predicts restrictive and repetitive behaviors and social cognition, working memory and monitoring skills predict social communication and initiation predicts social motivation [32]. This topic entails evident complexity that is accentuated by the developmental effect; behavioral regulation abilities are more correlated to social responsiveness in children with ASD, while metacognitive abilities remain significantly correlated to social skills both in childhood and adolescence [33].

In conclusion, adolescents with ASD without ID show greater difficulties in social communication abilities, EF, and ToM than expected for their cognitive level. Longitudinal studies are needed to gain further understanding of the long-term impact of different symptom severity profiles established in childhood ASD without ID once they reach adolescence, and to explore whether these empirically derived subgroups evolve differently over time. To fill this gap, the present study was designed to address the following objectives: (1) to identify empirically, through cluster analysis, subgroups of individuals with ASD without ID based on the severity of the core symptoms in childhood; (2) to explore whether these subgroups of ASD without ID and a group with typical development differ in cognitive, adaptive, and social aspects measured in adolescence. Outcome measures included IQ, EF, ToM, prosocial behavior, and difficulties with peer relationships, everyday life skills, and socialization and social integration in school. 

## 2. Materials and Methods

### 2.1. Participants

The sample at baseline assessment included 52 children with autism without intellectual disability (ID) and 37 children with typical development (TD). The two groups of children were between 7 and 11 years old, and their intellectual functioning was within the normal range as per the scores on the Kaufman Brief Intelligence Test (K-BIT) [34]. Both groups of children, with ASD and with TD, were matched on age (t(87) = −0.15, *p* = 0.88), IQ (t(87) = −0.28, *p* = 0.78), and their level on the vocabulary subtest from the Wechsler Intelligence Scale for Children (WISC-IV) [35] (t(87) = 1.89 *p* = 0.06).

The clinical group had received a clinical diagnosis of ASD in child and adolescent mental health and neurology services in the Valencian Community, according to the DSM-IV criteria [4], the ADI-R [3], and/or the ADOS [2]. Additionally, a clinical psychologist from the research team administered the ADI-R and Social Communication Questionnaire (SCQ) [36] again to the parents, following the recommended cut-off points for ASD diagnosis. All the children also met diagnostic criteria for ASD from the DSM-5 [1], based on information reported by teachers and parents. Both informants, through interviews with the research team, rated the severity of the criteria in the two ASD dimensions on scales ranging from 0 to 3 points (0 represents ‘almost never’, 1 ‘sometimes’, 2 ‘often’, and 3 ‘many times’). The Kappa-Cohen test value was κ = 0.93.

Exclusion criteria, evaluated through an extensive anamnesis carried out with the families, were as follows: any neurological or genetic diseases, brain lesions, sensory, auditory, or motor deficits, and an IQ below 80. Associated psychopathological problems, such as language disorders, learning disabilities, anxiety and mood disorders, or behavioral problems were not considered exclusion criteria.

On average, five years after baseline, the follow-up included 72 adolescents between 12 and 15 years old: 45 adolescents with ASD without ID (retention rate of 86.5%) and 27 adolescents with TD (retention rate of 72.9%). The sample loss was due to various factors: families could not be contacted, families had relocated, or declined the invitation to attend the evaluations. In the clinical group, there were no differences between the IQ and ASD symptom severity of the 45 children who continued in the study and the seven children who did not, based on parental ratings on the Strengths and Difficulties Questionnaire (SDQ) [37] and the DSM-5 diagnostic criteria [1]. At follow-up, both groups of adolescents, those with ASD and those with TD, were matched on age (t(70) = 0.65, *p* = 0.51), IQ (t(70) = −0.13, *p* = 0.89), and vocabulary (t(70) = 1.18, *p* = 0.24). 

Additionally, 77.7% of the adolescents with ASD were receiving educational support; 20 adolescents (44.4%) attended mainstream classrooms but received educational support for their specific needs in their high school; and 15 adolescents (33.3%) were placed in the communication and language classroom modality. Finally, ten adolescents with ASD (22.2%) were attending high school in regular classrooms full time without educational support. Regarding psychiatric medication, 40% of the adolescents with ASD were taking antipsychotic medication (mostly risperidone) and/or methylphenidate for behavioral problems and irritability symptoms (see Table 1). 

The neurotypical participants were in the same schools as the clinical sample in the study. They had no history of psychiatric disorders or had ever been referred to child and adolescent mental health services and they did not meet DSM-5 [1] criteria for ASD on the screening carried out before beginning the evaluation. Table 1 shows descriptive characteristics of the participants in both groups, ASD and TD.

### 2.2. Measures

#### 2.2.1. Time 1. Severity of ASD Symptom Measures

Social Communication Questionnaire (SCQ) [36]. This parental reported questionnaire provides information about three domains of autistic symptoms: reciprocal social interaction, social communication, and restricted, repetitive behaviors. The SCQ [36] does not exclusively assess DSM criteria for ASD. However, the items refer to behaviors that are specific examples of these criteria in order to improve parents’ understanding. The items have two response options (absence = 0; presence = 1), with higher scores indicating greater severity. In the present study, the Cronbach’s alpha for the SCQ [36] was 0.78, similar to what was reported by Rutter et al. (2003).

DSM-5 criteria for ASD [1]. Parents were asked to rate behaviors referring directly to blocks A and B of the DSM-5 [1] criteria for autism behaviors on a scale with four response options (never = 0, sometimes = 1, often = 2, very often = 3): A. Persistent deficits in social communication and social interaction across contexts, not accounted for by general developmental delays; B. Restricted, repetitive patterns of behavior, interests, or activities. 

Socio-demographic interview. Socio-demographic information was collected in semi-structured interviews with parents, and included: parents’ and child’s gender, age, mother’s marital status, mother’s and father’s education level, parents’ history of mental health, family structure variables, as well as the child’s characteristics (i.e., age, gender, child’s developmental history, age of diagnosis, medication, academic performance, support received in school).

#### 2.2.2. Time 2. Follow-up Measures. Cognitive Outcomes

Executive Functioning (EF). The teacher’s version of the Behavior Rating Inventory of Executive Functions (BRIEF) [38] was applied to assess the child’s EF through the teacher’s observations of his/her behavior in the school context. It consists of 86 items rated on a three-point Likert-type scale (never, sometimes, often). The items are grouped into 8 scales: inhibit; shift; emotional control; initiate; working memory; plan/organize; organization of materials; and monitor. Direct scores can be transformed into T-scores, with higher scores indicating worse EF. The questionnaire’s reliability and validity have been adequately demonstrated [38] and have been confirmed in the Spanish population (Cronbach’s α = 0.90 to 0.56 between subscales) [39].

#### 2.2.3. Time 2. Follow-up Measures. Socio-Adaptive Outcomes

Social Cognition. Applied Theory of Mind was assessed based on information provided by the parents on the advanced subscale of the Theory of Mind Inventory (ToMI) [40,41]. The advanced subscale (ToMI-A) is composed of 16 items that assess more mature aspects of ToM: second-order beliefs and competence in making inferences and complex social judgments. Each item is rated from 0 to 20, ranging from ‘definitely not’ to ‘definitely’, with a midpoint of ‘undecided.’ Higher scores indicate the perception of good ToM development across the range of content surveyed. The ToMI has been sufficiently validated and has good internal consistency and test–retest reliability, as well as excellent sensitivity (0.90) and specificity (0.90) [40,41].

Social Behavior. The SDQ [37], filled out by parents, contains a total of 25 items with three response options (0 = not at all true; 1 = somewhat true; 2 = very true). The items are grouped into five subscales: hyperactivity/attention problems, emotional problems, behavioral problems, peer relationship problems, and prosocial behavior. In this study, we used the peer relationship problems and prosocial behavior subscales. Higher scores on the peer relationship problems subscale indicate a greater likelihood of significant problems, whereas the prosocial subscale provides a reverse score where higher scores indicate more prosocial behaviors or strengths. The SDQ has shown good statistical and psychometric properties, with Cronbach’s alpha values above 0.70 [42], confirmed in the Spanish population (0.76) [43]. 

Social Competence in School Setting. The peer relations subscale, which is included in the School Social Behavior Scales (SSBS) [44], was filled out by teachers. It is composed of 14 items that are rated by the teacher on a five-point Likert-type scale (from never to very often). The items measure social skills or characteristics that are important in establishing positive relationships with and gaining social acceptance from peers at school. SSBS have demonstrated satisfactory internal consistency (0.98), test–retest reliability (ranging from 0.60 to 0.82), and convergent and discriminant validity with other behavior rating scales [44]. Cronbach’s alpha in our sample of the peer relationship subscale is 0.71. 

Adaptive Behavior. Socialization and daily living skills were evaluated with the Vineland Adaptive Behavior Scale (VABS-II) [45], a semi-structured interview for parents. The daily living domain describes skills related to personal (e.g., eating, dressing, hygiene), domestic (e.g., household tasks performed), and community (e.g., using money, answering the phone) tasks. The socialization domain includes skills related to interpersonal relationships, play and leisure, and coping skills. The VABS has been widely used in clinical, educational, and research settings with high functioning individuals with ASD [46]. It has solid psychometric properties, with high test–retest reliability (α = 0.98) [46].

Transition Success to Secondary Education [47,48]. Parents filled out a questionnaire answering six questions related to three factors. Regarding the ‘developing friendships and confidence’ dimension, parents were asked whether, compared to primary school, their child has more (3), the same number (2), or fewer (1) school friends, as well as more, the same, or less self-esteem, confidence, and motivation. The same scoring system was used in the ‘experiencing curriculum continuity’ dimension, which was assessed by asking parents whether, compared to primary school, their child shows more, the same, or less interest in school and schoolwork. Regarding the ‘adaptation to school life’ dimension, parents were asked how they thought their child had settled in (very well, 4; fairly well, 3; not very well, 2; not well at all, 1). They were also asked how satisfied they were with the whole process of their child’s transition to secondary school (from very satisfied, 4 points, to not at all satisfied, 1 point), how they felt when their child first moved on to secondary school, and how they feel now (from not at all concerned, 4 points, to very concerned, 1 point). The index of internal consistency Cronbach’s alpha in our sample is 0.91.

### 2.3. Procedure

The follow-up assessment was carried out in the high schools where the participants were enrolled, in spaces with optimal conditions for psychoeducational assessment. The information about executive functioning was collected through questionnaires filled out by teachers/tutors. The parents (mostly mothers and sometimes both parents) provided information about their children’s ToM skills in daily life contexts, ASD symptoms, and adaptive/social skills. The assessment was performed by ASD experts trained in the administration and rating of the questionnaires used. All the participants gave their written consent after being informed of the study goals. All the procedures were performed following the ethical standards of the Ethics Committee of the University of Valencia (Procedure number H1425284258543), according to the principles of the Helsinki Declaration of the World Medical Association (WMA) [49]. Likewise, the present follow-up study received authorization from the Board of Education of the Valencian Government.

### 2.4. Statistical Analysis

The statistical analyses were performed with the Statistical Package for the Social Sciences (SPSS v 26.0. IBM, Barcelona, Spain 2012). The distribution of the variables was analyzed, as well as their fit to the normal distribution curve, by applying the Kolmogorov–Smirnov test, transforming only the variables that showed an anomalous distribution using natural log transformation.

A model-based cluster analysis was performed to examine distinct profiles/groups at baseline, based on the severity of the autism symptoms. The input for this analysis included criteria A and B from the DSM-5 (APA) [1] and the three SCQ scales [36], which are used to measure core ASD symptoms and could be reliable means of rating severity. Both instruments focus on social communication ability and understanding and restricted and repetitive patterns of behavior.

The first step was to determine the optimal number of clusters. For this purpose, hierarchical cluster analysis was performed using squared Euclidean distance measures and Ward’s minimum variance method to establish homogenous cases because this type of solution is more appropriate for small samples [50]. Ward’s method is a minimum variance procedure used for hierarchical cluster analysis that has been found to be preferable to other methods, such as the single-link method. This method demonstrates a strong sensitivity to outliers and a tendency to suggest clusters that are similar in size [51]. For these procedures, the variables were standardized to z-scores. 

The next step was to use k-means analysis because this procedure allows us to specify the number of clusters in advance. Moreover, to fit the optimal cluster analysis solution, we used the variance ratio criterion (VRC) for each selected cluster. The VRC refers to the ratio of ‘within variance’ (variance explained by the typology) and ‘between variance’, corrected for the number of clusters and responses. A three-cluster solution seemed to be optimal in the hierarchical cluster analysis based on Ward’s method. It also seemed to be a meaningful solution based on visual inspection of the dendrogram figure and the agglomeration coefficients [52]. Additionally, external validity was established by conducting ANOVAs using variables that were not included in the hierarchical cluster [53]. In the current study, a one-way multivariate analysis of variance (MANOVA) was used to establish the external validity of the selected clusters by comparing the groups’ standardized scores with subdomain scores from two scales of the Children’s Communication Checklist (CCC-2) [54], designed to obtain information about autistic features that are not directly related to language (social relationships and interests). Cluster solutions were interpreted as low, moderate, and high autism core symptom severity. 

Finally, one-way multivariate analyses of variance (MANOVAs) with Tukey post hoc analyses were conducted to determine the effects of differences in autism symptom severity in the three clusters at baseline on cognitive, social, and adaptive outcomes at follow-up. Cognitive outcomes: IQ and BRIEF; Social outcomes and adaptive skills: peer problems and prosocial scale (SDQ); School Social Behavior (SSBS); Advanced scale of ToMI, the EPPSE transitions questionnaire, and two scales of daily life skills and socialization (VABS-II). The proportion of total variance accounted for by the independent variables was calculated using partial eta squared, according to Cohen [55]: eta squared, 0.06 = small; 0.06–0.14 = medium, 0.14 = large. 

## 3. Results

### 3.1. Subgroups of Children with ASD without ID Based on the Severity of the Autism Symptoms at Baseline

The results of the hierarchical cluster analysis based on the severity of the autism symptoms at baseline determined an optimal number of clusters in three groupings, distinguished by the tendency of their scores on the variables included in the analysis: DSM-5 criteria A (social communication impairment, SCI), DSM-5 criteria B (restricted and repetitive patterns of behavior, RRBI), and SCQ subscales (social deficits, communication abnormalities, and RRBI). Cluster 1 (*n* = 21; 40.3%) presented higher autism symptom severity scores on both the DSM-5 and SCQ, and it was labeled ‘high severity’. Cluster 2 (*n* = 18; 34.6%) showed an intermediate position on most of the variables, and it was considered the ‘moderate severity’ group on most of the scores. Cluster 3 (*n* = 13; 25.0%) showed lower scores than Clusters 1 and 2 on most of the variables analyzed, and so it was classified as the ‘low RRBI severity’ group. 

Multivariate Analysis of Variance (MANOVA) was conducted to determine the significant differences between the three clusters on the severity of the autism symptoms. The MANOVA conducted to assess the main group effect among the three groups was statistically significant (Wilk‘s Lambda (Λ) = 0.07, F_(10,90)_ = 23.38, *p* < 0.001, η^2^p = 0.72). ANOVAs revealed significant differences in DSM-5 criteria A (social communication ability), DSM-5 criteria B (restricted and repetitive patterns of behavior), and the SCQ subscales (social deficits, communication abnormalities, and restricted, repetitive, and stereotyped behavior). The effect sizes of the differences between clusters had high values, although the severity and extension varied (see Table 2).

**Figure 1:**
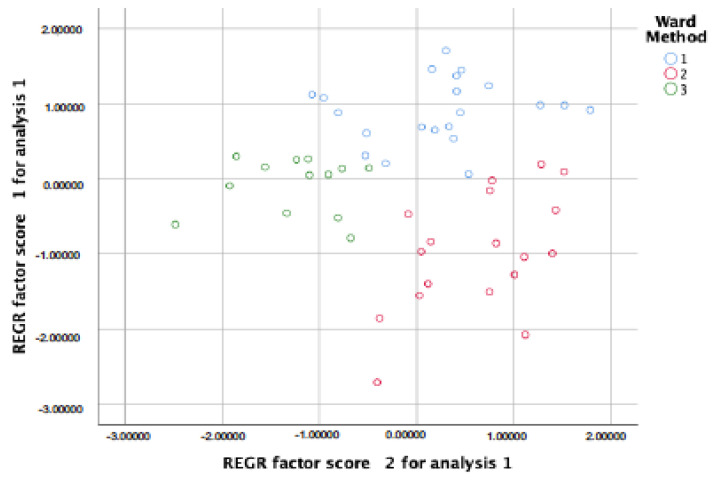
Scatterplot of pairwise comparisons between clusters 1 (blue), 2 (red), 3 (green) on ASD without ID DSM-5 and SCQ subscales.

Tukey post hoc analyses showed statistically significant differences on DSM-5 criteria A (social communication ability) between Cluster 1 ‘high severity’ and Cluster 2 ‘moderate severity’, whereas there were no significant differences between Cluster 1 and Cluster 2 on DSM-5 criteria B (RRBI) and the SCQ restricted, repetitive, and stereotyped behavior subscale, although both clusters showed significant differences with Cluster 3 ‘low RRBI severity’. A similar pattern was observed on the SCQ subscales: on social deficits and communication problems, there were significant differences among the three clusters, but there were no statistically significant differences between Cluster 2 ‘moderate severity’ and Cluster 3 ‘low RRBI severity’ on the SCQ social deficits scale (Table 2 and Figure 1). The scatterplot (Figure 1) helped us visualize how sets of data are related. REGR scores represent the coefficient of regression equation for each cluster.

### 3.2. Differences in Cognitive Outcomes at Follow-up Across the Subgroups of Adolescents with ASD without ID and Adolescents with TD

**Figure 2:**
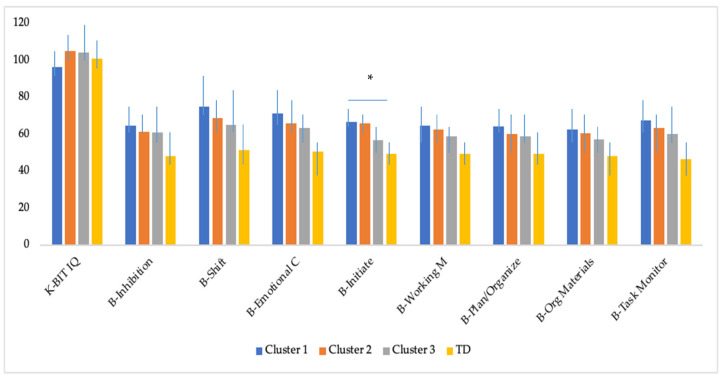
Cognitive outcomes of all four on K-BIT and BRIEF. Graphs represent means with standard deviation bars. Differences between clinical groups (Cluster 1 high severity, Cluster 2 moderate severity, Cluster 3 low severity and TD) are highlighted, * *p*-values < 0.05.

Table 3 presents the comparison of the three clusters of ASD, ‘high severity’, ‘moderate severity’, ‘low severity’, and the typical development group on cognitive outcomes at follow-up. The MANOVA conducted to assess the main group effect among the four groups was statistically significant (Wilk‘s Lambda (Λ) = 0.33 F_(27,175)_ = 3.01, *p* < 0.001, η^2^p = 0.31). The analysis of variance revealed statistically significant differences between the groups in all the executive functioning variables (BRIEF) included. After applying the Bonferroni correction (*p* < 0.005), the variables that remained significant were the same. Tukey post hoc analyses showed statistically significant differences between TD and the three clusters on inhibition, shift, initiate, and task monitor. Moreover, there were significant differences between TD and Cluster 1 ‘high severity’ and Cluster 2 ‘moderate severity’ on emotional control, working memory, plan/organize, organization of materials, and initiate, whereas there were no significant differences in these variables between TD and Cluster 3, ‘low RRBI severity’. Lastly, results revealed statistically significant differences between Cluster 1 and Cluster 3 (*p* = 0.009) and between Cluster 2 and Cluster 3 (*p* = 0.018) on initiate. Scores obtained by the four groups are illustrated in Figure 2.

### 3.3. Differences in Social and Adaptive skills at Follow-up Across the Subgroups of Adolescents with ASD without ID and Adolescents with TD

**Figure 3:**
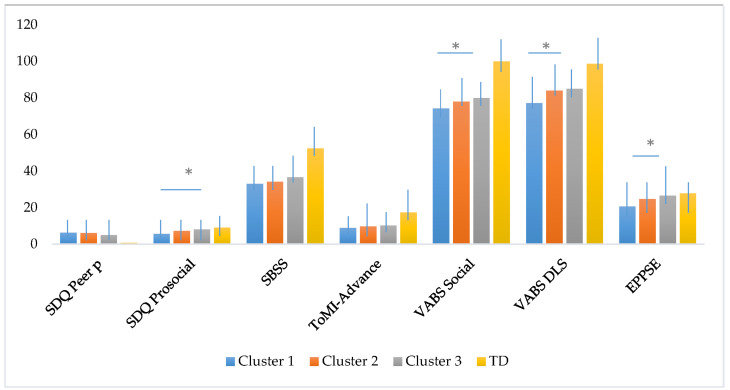
Social, cognitive and adaptive skills of the four groups. Graphs represent means with standard deviation bars. Differences between clinical groups (Cluster 1 high severity, Cluster 2 moderate severity, Cluster 3 low severity and TD) are highlighted, * *p*-values < 0.05.

Table 4 presents the comparison of the three clusters of ASD, ‘high severity’, ‘moderate severity’, ‘low RRBI severity’, and the typical development group on social outcomes, social cognition, and adaptive skills at follow-up. The MANOVA conducted to assess the main group effect among the four groups was statistically significant (Wilk‘s Lambda (Λ) = 0.62 F_(21,175)_ = 13.6, *p* < 0.001, η^2^p = 0.60). The analysis of variance revealed statistically significant differences between the groups on all the variables included. After applying Bonferroni correction (*p* < 0.007), the variables that remained significant were the same. Tukey post hoc analyses showed statistically significant differences between TD and the three clusters on peer problems (SDQ) SBSS, ToMI Advanced, social domain, and daily life skills (VABS). Moreover, there were significant differences between Cluster 1, ‘high severity’, and Cluster 2, ‘moderate severity’, and Cluster 3, ‘low RRBI severity’, on daily life skills and socialization. The prosocial scale (SDQ) showed significant differences between TD and Cluster 1 and Cluster 2 and between Cluster 1 and Cluster 3, whereas the EPPSE scale showed significant differences between TD and Cluster 1, and between Cluster 1 and Cluster 3. Scores obtained by the four groups are illustrated in Figure 3.

Furthermore, in the follow-up, 13 participants failed to meet ASD diagnostic criteria on the SCQ and the DSM-5. Specifically, the adolescent with remitted diagnostic ASD symptoms according to both measures were: three in Cluster 1 (16.67%), six in Cluster 2 (37.5%), and four in Cluster 3 (36.4%).

## 4. Discussion

The present study had two objectives. The first was to identify subgroups of children with ASD without ID, based on the severity of the core symptoms, and explore their impact on EF and social and adaptive skills in adolescence. Regarding the first aim, cluster analysis revealed the presence of three groups. Cluster 1 (40.4%), ‘high severity’, showed higher intensity and frequency of autism features, whilst Cluster 2 (34.6%), ‘moderate severity’, and Cluster 3 presented similar levels of social impairment. However, Cluster 3 (25%), ‘low severity’, showed significantly lower scores on the RRBI and the communication abnormalities subscale of the SCQ. Our findings are consistent with other studies with samples of children with ASD with intellectual deficits or heterogeneity in their cognitive levels. Specifically, Cholenkery et al. [5] identified three clusters: one had low scores on autism features; another one included individuals with a high presence of autistic traits; and a third cluster was characterized by social and communicative impairments and less RRBI. Georgiades et al. [6], using data on social communication deficits and fixated interests and repetitive behaviors, differentiated three subgroups with similar characteristics and numbers of participants. Greater SCI and RRBI corresponded to the most numerous subgroup (56%), whereas fewer participants (10%) made up the subgroup with less SCI and relatively high scores on RRBI. Moreover, based on the levels of RRBI, rather than on the social aspects of autism, Zheng et al. [7] reported three subgroups of children with ASD without ID. In parallel, all the subgroups differed on an extended spectrum of clinical behavioral and adaptive measures. Likewise, in this subtyping study, RRBI provided useful information to differentiate between two subgroups of ASD with moderate–severe SCI and characterize clinical phenotypes in autism without ID. 

The second objective was to explore possible differences in cognitive, social, and adaptive aspects in adolescents with ASD without ID and TD adolescents. This study extends previous longitudinal studies of clusters by focusing on adolescence, a particularly complex developmental period. Regarding the extent to which the subgroups differed on their EF trajectories over time, significant differences and moderate effect sizes were observed for most subdomains, as measured with the BRIEF, in individuals with ASD without ID when compared to TD. Specifically, all three subgroups of ASD had more significant problems with inhibition, shift, initiate, and task monitor than the TD group, even though they were matched on cognitive ability. These findings show that EF may be a potential endophenotype in the investigation of possible subtypes within the spectrum [56]. The diversity in the specific executive impairments related to symptom severity should also be highlighted. Ratings on emotional control, working memory, planning, and organization of materials from the BRIEF of two of the subgroups, ‘high severity’ and ‘moderate severity’ on the SCI and RRBI, were significantly lower than for TD participants. The difficulties noted in these areas of EF in the ‘low RRBI severity’ subgroup were also greater than in the TD group, although without reaching statistical significance, hence indirectly supporting the reported relationship between poorer EF and greater severity of ASD symptoms found in other empirical studies [57]. 

The exploration of social and adaptive trajectories among the three clusters of ASD without ID yielded interesting findings. On the one hand, regardless of the level of ASD symptom severity, all the subgroups presented significant differences in the majority of the aspects assessed, namely, more difficulties in peer relationships and in daily living skills, along with poor ToM skills and low social competence in school settings. Adolescents with ASD, even when they are cognitively able, and regardless of the symptom severity during childhood, generally present deficits in social and adaptive skills. On the other hand, the developmental, social, and adaptive trajectories of ASD without ID subgroups were heterogeneous in some ways. Thus, the subgroup with ‘high severity’ of SCI and RRBI showed adaptive behavior that was significantly worse in the socialization and daily living skills domains than the other two ASD subgroups, ‘moderate severity’ and ‘low RRBI severity’. Moreover, prosocial behavior, such as cooperating, sharing, expressing empathy, or providing emotional support, was expressed differently quantitatively, so that no significant differences between TD and ‘low RRBI severity’ were noted, unlike the ‘high severity’ subgroup. 

Precisely, the joint analyses of the results obtained in executive functions and social-adaptive behaviors in the present study indicated that Cluster 3 (‘low severity’) showed a significantly higher level of initiative than the other two clusters (‘moderate’ and ‘high severity’). This suggests that a greater executive functioning, and particularly initiative, has a positive impact on prosocial behavior and optimal academic adaptation [32]. 

In summary, this prospective study highlights that children with ASD without ID may be classified into three subgroups differentiated through cluster analysis, based on the severity of symptoms in dimensions of SCI and RRBI. Although the magnitude of the follow-up results in adolescence should be interpreted with caution, the subgroups identified showed relatively different developmental trajectories that were especially evident in the case of socialization, daily living skills, and prosocial behavior, and much more limited in EF. Unfortunately, the few previous prospective studies of ASD clusters [16,17,18] have different characteristics (e.g., in IQ, participants’ age, time of follow-up, measures used) from those of the present study, which limits the comparison of the findings. 

Our research is exploratory and has some limitations related to the characteristics of the sample and the evaluation procedure. First, the relatively small sample size may be hiding some possible significant relationships between the study variables. However, our sample provided a stable clustering solution. Nevertheless, to increase the power for differentiating the subgroups, future studies should increase the number of participants using randomized sampling. The second limitation is that the participants had a mid-range intellectual capacity, and most of them were male, which may affect the generalization of the results. The findings may not apply to girls or individuals with ASD with other cognitive levels. Another limitation was that our study was unable to examine the potential effects of interventions received earlier in life, that is, between the baseline and follow-up evaluations. Participants received a wide variety of pharmacological and psychoeducational interventions during this time; however, these data were not available with the necessary systematization to include them in our analyses. Likewise, potentially important modifiable protective factors of the family (e.g., parental coping strategies) or school (e.g., level of inclusion) context were not taken into account. Another limitation is related to the assessment method. The information provided may have been affected by errors because it relies on parent reports. The questionnaires used, despite having good psychometric properties, can be affected by different types of biases, such as social desirability or recall errors. The results should be replicated with larger samples and assessment procedures with less chance of bias.

Despite the limitations, the present study extends previous research in the field and has important clinical implications. It used a wide range of assessment measures covering specific cognitive, adaptive, and social domains, and it analyzes the subgroups’ EF and socio-personal adaptation in adolescence, a stage that has received little attention. Broadening our knowledge on the role of executive functioning in ASD longitudinal outcomes could contribute to improving assessment and intervention in daily clinical practice. 

A practical implication of the study is that ASD without ID children with greater symptom severity were found to be more vulnerable to experiencing adaptive difficulties in adolescence. More attention should be paid to assessing the role of early intensive interventions in enhancing social and adaptive skills. Another implication of our findings is the relevance of restricted and repetitive behavior as a core feature of ASD. This heterogeneous set of behaviors—intense preoccupation, stereotyped movements, and resistance to change [1]—poses a major challenge to the executive, social, and adaptive development of individuals with ASD, even those who are cognitively able. Although the results are still quite limited [58], reducing RRBI should be a key objective in treatments for ASD. 

## Figures and Tables

**Table 1:** Socio-demographic characteristics of the study sample.

	Baseline			Follow-up		
Participants	ASD (*n* = 52)	TD (*n* = 37)	t/χ^2^	ASD (*n* = 45)	TD (*n* = 27)	t/χ^2^
Age	8.6 (1.3)	8.5 (1.2)	−0.15	12.9 (0.9)	12.7(1.0)	0.65
Full IQ	101.4 (12.6)	102.1 (8.9)	0.28	101.5 (12.9)	101.1 (8.2)	−0.13
Vocabulary	11.7 (2.7)	12.9 (2.7)	1.89	11.4 (2.9)	12.3 (3.1)	1.18
SCQ-Total	22.9 (6.5)	3.1 (2.7)	−17.4 *	14.2 (5.2)	2.6 (1.6)	−11.0 *
ADI-R A	13.4 (2.7)					
ADI-R B	8.9 (2.4)					
ADI-R C	4.7 (1.9)					
Educational Support	96.1%	0.0%	81.1 *	77.7%	7.4%	33.4 *
Gender (% Males)	92.3%	62.1%	12.17 *	91.1%	66.6%	6.81 *
Medication (% yes)	32.7%	0.0%	14.9 *	40.0%	0.0%	14.4 *

Data are presented as mean (standard deviation), * *p*-values < 0.05. Abbreviations: ADI-R A (qualitative alterations in the reciprocal social interaction), ADI-R B (qualitative alterations in communication), ADI-R C (restrictive and stereotyped behaviors), ASD (Autism Spectrum Disorder), SCQ (Social Communication Questionnaire), TD (Typical Development).

**Table 2:** Analyses of variance (ANOVAs) for the severity of autism symptoms at baseline.

ASD Symptom Severity	Cluster 1 (*n* = 21) High Severity	Cluster 2 (*n* = 18) Moderate Severity	Cluster 3 (*n* = 13) Low RRBI Severity	F_(2,49)_	*p*	η^2^p	Tukey Post Hoc
DSM-5 criteria A	7.8 (1.1)	6.5 (1.9)	6.6 (1.1)	4.26	0.02 *	0.15	1 > 2
DSM-5 criteria B	9.9 (1.6)	9.1 (1.6)	7.1 (1.1)	13.98	0.00 **	0.36	1, 2 > 3
SCQ Social	13.1 (2.1)	7.0 (3.2)	8.3 (1.6)	32.90	0.00 **	0.57	1 > 2, 3
SCQ Comm	9.5 (1.3)	5.7 (1.7)	7.0 (1.3)	32.60	0.00 **	0.57	1 > 2, 3; 2 < 3
SCQ RRS	5.4 (1.5)	5.9 (1.1)	2.4 (1.3)	28.18	0.00 **	0.53	1, 2 > 3

Data are presented as means (standard deviations), * *p*-values < 0.05, ** *p*-values < 0.01. Abbreviations: DSM-5 criteria A (social communication ability), DSM-5 criteria B (restricted and repetitive patterns of behavior), SCQ Social (Social Communication Questionnaire, social deficits), SCQ Comm (communication abnormalities), and SCQ RRBI (restricted, repetitive, and stereotyped behavior).

**Table 3:** Analyses of variance (ANOVAs) for cognitive outcomes at follow-up.

Cognitive Outcomes	Cluster 1 (*n* = 18) High Severity	Cluster 2 (*n* = 16) Moderate Severity	Cluster 3 (*n* = 11) Low RRBI Severity	TD (*n* = 27)	F_(3,68)_	*p*	η^2^p	Tukey Post Hoc
K-BIT IQ	96.3 (10.5)	105.3 (13.0)	104.4 (14.3)	101.1 (8.2)	2.21	0.09	0.08	n.s.
B-Inhibition	64.8 (16.0)	61.5 (17.2)	60.8 (13.5)	48.1 (4.5)	7.45	0.00 *	0.24	TD < 1, 2, 3
B-Shift	75.1 (14.0)	68.8 (16.1)	64.9 (13.3)	51.4 (7.6)	14.61	0.00 *	0.39	TD < 1, 2, 3
B-Emotional C	71.3 (20.8)	65.8 (19.3)	63.4 (16.8)	50.5 (6.7)	7.07	0.00 *	0.23	TD < 1, 2
B-Initiate	66.6 (9.4)	66.1 (7.8)	57.0 (7.2)	49.3 (5.9)	25.57	0.00 *	0.53	TD < 1, 2, 3; 3 < 1, 2
B-Working M	64.6 (12.9)	62.5 (10.7)	58.9 (13.9)	49.3 (6.0)	9.51	0.00 *	0.29	TD < 1, 2
B-Plan/Organize	64.3 (13.2)	60.1 (10.1)	58.7 (12.8)	49.2 (5.0)	9.17	0.00 *	0.28	TD < 1, 2
B-Org Materials	62.5 (16.4)	60.7 (14.5)	57.1 (14.8)	48.2 (6.3)	5.65	0.00 *	0.20	TD < 1, 2
B-Task Monitor	67.4 (12.9)	63.5 (13.1)	60.1 (12.2)	46.6 (5.8)	16.19	0.00 *	0.41	TD < 1, 2, 3

Data are presented as means (standard deviations), * *p*-values < 0.005 (Bonferroni correction). Abbreviations: B (BRIEF—Behavior Rating Inventory of Executive Function), Emotional C (emotional control), Working M (working memory), Org Materials (organization of materials), n.s. (non-significant).

**Table 4:** Analyses of variance (ANOVAs) for social outcomes, socio-cognitive skills, and adaptive behavior at follow-up.

Socio-Adaptive Outcomes	Cluster 1 (*n* = 18) High Severity	Cluster 2 (*n* = 16) Moderate Severity	Cluster 3 (*n* = 11) Low RRBI Severity	TD (*n* = 27)	F_(3,67)_	*p*	η^2^p	Tukey Post Hoc
Peer Prob (SDQ)	6.2 (1.4)	6.1 (2.0)	4.9 (1.5)	0.7 (1.0)	67.65	0.00 *	0.75	TD < 1, 2, 3
Prosocial (SDQ)	5.6 (2.5)	7.3 (1.9)	8.0 (1.5)	9.0 (1.1)	12.51	0.00 *	0.35	TD > 1, 2; 1 < 3
SBSS	33.0 (6.8)	34.1 (11.2)	36.5 (8.9)	52.3 (7.3)	25.55	0.00 *	0.53	TD > 1, 2, 3
ToMI-Advanced	8.9 (3.5)	9.7 (3.5)	10.1 (2.6)	17.4 (1.7)	54.65	0.00 *	0.71	TD > 1, 2, 3
Social (VABS)	74.1 (5.1)	78.0 (3.0)	79.9 (7.0)	100 (10.1)	61.97	0.00 *	0.73	TD > 1, 2, 3; 3, 2 > 1
Daily LS (VABS)	77.1 (3.9)	84.0 (5.3)	84.9 (6.0)	98.6 (5.9)	54.79	0.00 *	0.71	TD > 1, 2, 3; 3, 2 > 1
EPPSE	20.5 (5.4)	24.6 (5.7)	26.4 (6.3)	27.7 (3.8)	7.25	0.00 *	0.24	TD > 1; 3 > 1

Data are presented as means (standard deviations), * *p*-values < 0.007 (Bonferroni correction). Abbreviations: Peer Prob SDQ (Strengths and Difficulties Questionnaire; peer problems), SBSS (School Social Behavior Scales), ToMI-Advanced (Advanced Scale of Theory of Mind Inventory), Social VABS (Vineland Adaptive Behavior Scales, socialization skills), Daily LS VABS (daily life skills), EPPSE (Effective Preschool, Primary, and Secondary Education transitions).

## Data Availability

The datasets generated for this study are available on request to the corresponding author.
